# Defining a critical period in calvarial development for Hedgehog pathway antagonist-induced frontal bone dysplasia in mice

**DOI:** 10.1038/s41368-018-0040-z

**Published:** 2019-02-20

**Authors:** Yuanjing Jiang, Shixian Zhang, Chuanqing Mao, Yongzhen Lai, Di Wu, Hu Zhao, Caiyu Liao, Weihui Chen

**Affiliations:** 1 Department of Oral and Maxillofacial Surgery, Union Hospital, Fujian Medical University, Fuzhou, Fujian China; 2 Institute of Stomatology, Fujian Medical University, Fuzhou, Fujian China; 3 School and Hospital of Stomatology, Fujian Medical University, Fuzhou, Fujian China; 4 Department of Restorative Sciences, School of Dentistry, Texas A&M University, Dallas, TX USA; 5 Fujian Biological Materials Engineering and Technology Center of Stomatology, Fuzhou, Fujian China

**Keywords:** Bone development, Cell migration

## Abstract

The Hedgehog (Hh) signalling pathway is essential for cellular proliferation and differentiation during embryonic development. Gain and loss of function of Hh signalling are known to result in an array of craniofacial malformations. To determine the critical period for Hh pathway antagonist-induced frontal bone hypoplasia, we examined patterns of dysmorphology caused by Hh signalling inhibition. Pregnant mice received a single oral administration of Hh signalling inhibitor GDC-0449 at 100 mg•kg^−^^1^ or 150 mg•kg^−^^1^ body weight at preselected time points between embryonic days (E)8.5 and 12.5. The optimal teratogenic concentration of GDC-0449 was determined to be 150 mg•kg^−^^1^. Exposure between E9.5 and E10.5 induced frontal bone dysplasia, micrognathia and limb defects, with administration at E10.5 producing the most pronounced effects. This model showed decreased ossification of the frontal bone with downregulation of Hh signalling. The osteoid thickness of the frontal bone was significantly reduced. The amount of neural crest-derived frontal bone primordium was reduced after GDC-0449 exposure owing to a decreased rate of cell proliferation and increased cell death.

## Bone Development: Critical period for Hedgehog pathway in bone development

During embryonic development, the Hedgehog signalling pathway regulates the migration, proliferation and differentiation of cranial neural crest cells in the early frontal bone. The Hedgehog signalling pathway transmits information to embryonic cells for their proper cell differentiation, and increased or reduced function of that signalling results in various craniofacial malformations. A team headed by Weihui Chen at Fujian Medical University in China investigated the patterns of abnormalities caused by inhibition of Hedgehog signalling in pregnant mice at preselected embryonic time points. The team was able to identify the critical period for sensitivity to GDC-0449, a potent Hedgehog signalling inhibitor. The authors believe that their mouse model can be effective in further investigating the mechanisms of craniofacial malformations and will have a profound impact on identifying candidate human disease genes and associated environmental factors.

## Introduction

Craniofacial malformations are common congenital defects, accounting for more than three-quarters of all congenital malformations, and they require extensive medical intervention. They can be caused by a variety of stimulating factors during the period of embryonic development.^1^

The Hedgehog (Hh) signalling pathway plays a vital role in calvarial growth and patterning.^2,3^ In mammals, patterning and morphogenesis of the frontal bone, a major craniofacial structure, begins with the migration of frontal bone precursors derived from cranial neural crest cells (CNCCs) into the frontal bone primordium; in the mouse, this migration proceeds in a caudal-to-rostral direction beginning at E11.5, followed by apical migration at E13.5.^4^ The mesenchymal cells in the initial condensation then differentiate into osteoblasts. Meanwhile, preosteoblasts continue to proliferate to form the bone anlage. These osteoblasts within the primordium subsequently synthesize bone matrix through intramembranous ossification.^5,6^ Sonic Hedgehog (Shh) has been shown to be necessary in craniofacial and limb development. Deletion of Shh in mice causes holoprosencephaly characterized by encephalodysplasia and, in extreme cases, cyclopia, accompanied by other facial abnormalities and cleft palate.^7^ Indian Hedgehog (Ihh) is a critical regulator of endochondral ossification. Loss of Ihh decreases the proliferation of preosteoblasts, leading to a reduction of cranial bone size and widened cranial sutures. However, the mechanism underlying this phenotype remains unclear.^8,9^

GDC-0449 is a potent and selective Hh signalling inhibitor that blocks Hh signalling by binding to and inhibiting Smo.^10^ GDC-0449 is approved by the US Food and Drug Administration as a drug due to its promise as a treatment for advanced basal cell carcinoma.^11^ Single-dose pharmacokinetic assessment of GDC-0449 in mice has shown that its half-life and the percentage of the area under the fitted curve (AUC) increase with increasing dose, yielding a serum half-life of 25.3 h at 100 mg•kg^−1^.^12^ The potential adverse effects of GDC-0449 on the embryo/foetus have been investigated in other developmental toxicity tests. Pregnant mice that received GDC-0449 by gavage at doses of 50 mg•kg^−^^1^, 60 mg•kg^−^^1^, 80 mg•kg^−^^1^, 100 mg•kg^−^^1^ or 150 mg•kg^−^^1^ during gestation had phenotypes that included holoprosencephaly, clefts of the lip with or without cleft palate (CL/P), and clefts of the secondary palate only. Less attention has been paid to frontal bone defects.^13,14^

Both chemical and genetic disruptions of the Hh pathway have been proposed to result in frontal bone dysplasia. However, mounting research in this area has primarily utilized genetic abrogation, with little study of the perturbation of Hh signalling in vivo using exogenous inhibitors.^15^ Here we identify the function of Hh signalling in spatiotemporally dependent aspects of frontal bone development after in utero exposure to the natural Hh pathway antagonist GDC-0449 in the mouse. Our study reveals a critical period of sensitivity to GDC-0449 in frontal bone development of the mouse. We show that the Hh signalling pathway is critical for the migration, proliferation and differentiation of CNCCs during frontal bone development. The Hh signalling pathway controls osteogenesis by regulating the expression of Runx2. We also propose a tractable mouse model in which Hh signalling is implicated, which can be used to study frontal bone dysplasia, micrognathia and limb defects.

## Results

### Time-specific teratogenesis of Hh pathway inhibition

In preliminary experiments, pregnant mice were administered 60, 100, 150 or 200 mg•kg^−1^ body weight (b.w.) of GDC-0449 by oral gavage at preselected time points between E8.5 and E12.5. The animals that received 100 or 150 mg•kg^−^^1^ b.w. of GDC-0449 showed craniofacial defects, and embryos exposed to GDC-0449 at a dose of 150 mg•kg^−^^1^ b.w. exhibited an increased frequency of such defects. The dose of 60 mg•kg^−^^1^ b.w. was ineffective in causing craniofacial defects, and at the other extreme, maternal death occurred 100% of the time after exposure to GDC-0449 at 200 mg•kg^−^^1^ b.w. (data not shown). In this study, to further define the optimal teratogenic concentration of GDC-0449 and the critical period in which its administration is teratogenic, GDC-0449 was administered at a concentration of 100 or 150 mg•kg^−^^1^ b.w. by oral gavage to groups of pregnant mice at E8.5–E12.5. There was no maternal death or obvious embryonic lethality in any of the groups that received 100 mg•kg^−^^1^ b.w. Only when exposure to GDC-0449 at 150 mg•kg^−^^1^ b.w. occurred on E8.5 and E9.5 was there a significant increase in maternal death (2/5 and 3/6), and embryos showed maximal embryonic toxicity, with one-third of the foetuses being resorbed. However, exposure to GDC-0449 at 150 mg•kg^−^^1^ b.w. on E10.5, 11.5 or 12.5 did not produce these signs of toxicity. As is well known, teratogen exposure in the organogenetic period may easily result in an abnormal morphology and structure. However, teratogen exposure in earlier stages, such as during embryo vesicle formation, often leads to death of the embryo, and embryonic malformation in such cases is rare.^16^ Therefore, we suspect that exposure to GDC-0449 at high dosages during early stages of development may cause a high rate of embryonic lethality. Most interestingly, administration at 150 mg•kg^−^^1^ b.w. on E9.5 or 10.5 produced foetuses exhibiting craniofacial anomalies with complete penetrance (25/25 and 34/34, respectively) (Table 1).

**Table 1:** Craniofacial abnormalities of embryos exposed at different age stages to one of two concentrations of GDC-0449

Embryonic age	Pregnant females/*n*	Maternal lethality/*n*	Fetuses in total/*n*	Viable embryos/*n*	Stillbirths /*n*	Craniofacial anomalies /*n*	Ratio of affected fetuses /%
100mg•kg^−1^ b.w.							
E8.5	3	1	36	30	6	0	0
E9.5	3	0	40	38	2	6	15.8
E10.5	3	0	39	39	0	9	23.1
E11.5	3	0	40	40	0	6	15
E12.5	3	0	42	42	0	10	23.8
150mg•kg^−1^ b.w.							
E8.5	5	2	39	24	15	0	0
E9.5	6	3	38	25	13	25	100
E10.5	3	0	41	34	7	34	100
E11.5	3	0	40	38	2	16	42.1
E12.5	3	0	41	41	0	20	48.8

**Fig. 1:**
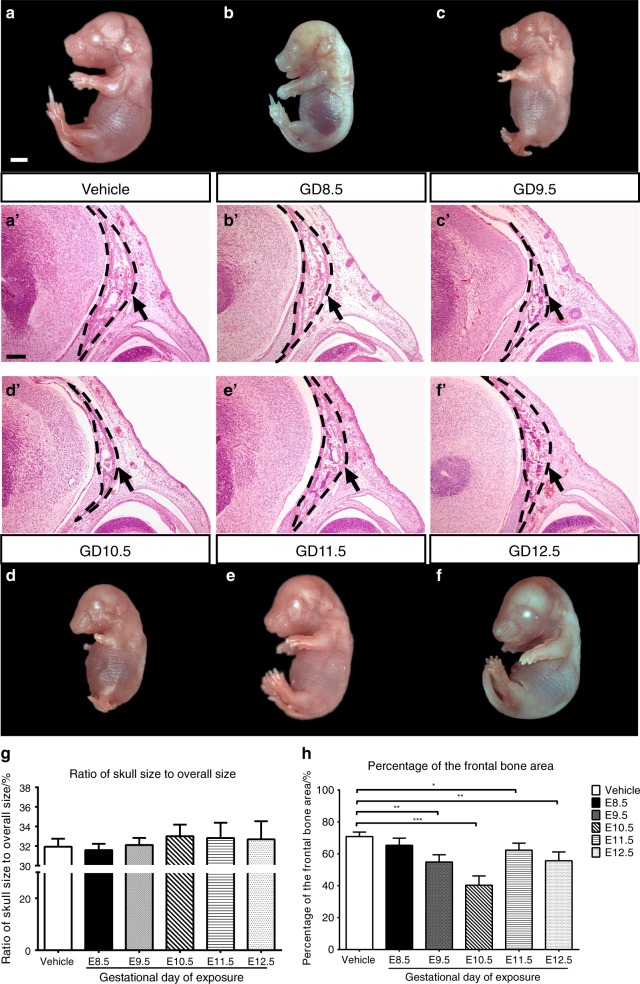
Stages of GDC-0449-induced facial dysmorphology in mouse embryos at E16.5. GDC-0449 was administered at 150 mg•kg^−^^1^ b.w. by gavage to groups of embryos at E8.5, 9.5, 10.5, 11.5 and 12.5 (henceforth, GD8.5-12.5 groups). **a**–**f** Analyses of mouse embryos under stereomicroscopy showed facial dysmorphology, including micrognathia and abnormal flexure at the skull and nasal juncture, which was especially obvious in the GD9.5 (**c**) and GD10.5 (**d**) groups. **a**'–**f**' H&E staining of vehicle-exposed and GD8.5-GD12.5 mice showing the frontal bone (arrow) formed by the trabecular bone (black dotted line), and a reduced bone matrix in the GD9.5 (**c**') and GD10.5 (**d**') groups. Scale bars = 1 mm, except 250 μm in (**a**'–**f**'). **g** Ratio of skull size to overall size. **h** Percentage of frontal bone area of the total frontal region. *n* = 6 embryos per group from three litters. Values are the mean ± SD. Groups were compared using one-way ANOVA. *P< 0.05; **P< 0.01; ***P< 0.001

**Fig. 2:**
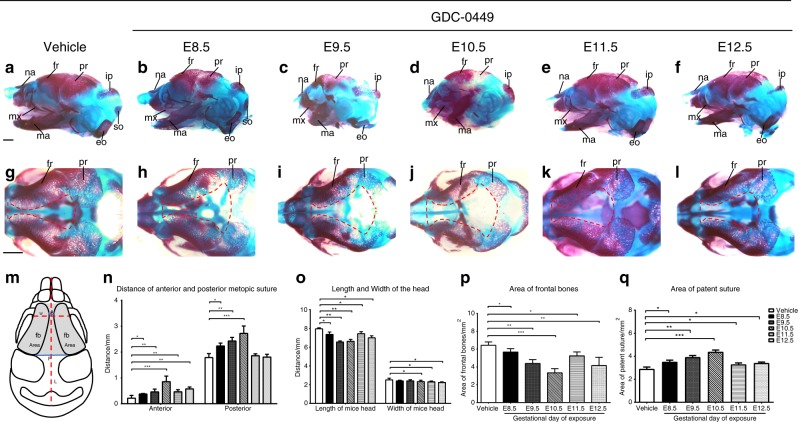
Exposure-dependent facial measurement analysis. Heads of embryos stained with Alcian blue and Alizarin Red S (red, bone; blue, cartilage). **a**–**f** Side views of skulls showing micromaxillary deformities and smaller skull bones in the GD8.5–GD12.5 groups. **g**–**l** Superior views of GD8.5-12.5 skulls showing gaps (red dotted lines) between the two frontal bones. In GD9.5 (**i**) and GD10.5 (**j**) embryos, the defect between the two frontal bones was significantly enlarged. **m** Schematic of a mouse head indicating the locations of the measurements. Comparison of the anterior metopic suture distance (A), posterior metopic suture distance (P), width of the head (W), length of the head (L), area of the frontal bones (fb), and area of the patent suture in the frontal region. Comparisons of vehicle-exposed and GDC-0449-exposed mice showing differences in **n** length of the anterior and posterior metopic suture, **o** length and width of the head, **p** area of the frontal bones and **q** area of the patent suture. (*n* = 6 embryos per group from three litters). The values are the mean ± SD. Groups were compared using one-way ANOVA. *P< 0.05; **P< 0.01; ***P< 0.001

We investigated the facial morphology of each group of embryos from dams that received 150 mg•kg^−^^1^ b.w. under a stereomicroscope, and there were no obvious differences in facial morphology in embryos exposed to GDC-0449 at E8.5, 11.5 or 12.5 (referred to henceforth as GD8.5 and so on) in comparison to the vehicle-exposed embryos (Fig. 1a, b, e, f). In contrast, in GD9.5 or 10.5 embryos, facial morphology was affected, producing significant abnormalities in the skull and mandible, as well as limb defects (Fig. 1c, d). Alcian blue and Alizarin Red S staining were performed to further characterize the different degrees of defects in the mandible, nasal, frontal and parietal bones. Moreover, a very large gap was detected between the frontal bones and parietal bones, especially in the GD9.5 and GD10.5 groups (Fig. 2a–l). In our histological analyses of sections of the frontal bones, we observed an obvious reduction in the expanse and thickness of the bone in GD9.5 and GD10.5 embryos compared with the vehicle-exposed controls (Fig. 1a'–f'). To assess variation in body size, we measured the ratio of skull size to overall size and the percentage of the frontal bone area of the total area of the frontal region (Fig. 1g, h). Although the overall size of the embryos was smaller in the GD9.5 and GD10.5 groups compared with the controls, there was no significant difference in the ratio of skull size to overall size between GDC-0449- and vehicle-exposed embryos. However, we found that the proportion of frontal bone in the frontal region of the skull decreased with stage-specific GDC-0449 exposure. Detailed facial quantitative analyses confirmed our observations in our subjective analyses using Alcian blue and Alizarin Red S staining (Fig. 2a–l). Compared with the vehicle-exposed embryos, the length of the anterior and posterior metopic suture increased, whereas the length and width of the head, area of the frontal bones, and area of the patent suture decreased with stage-specific GDC-0449 exposure. Relative to the GD8.5, 11.5 and 12.5 groups, the GD9.5 and GD10.5 groups showed the most significant differences. Taken together, these results suggested that the optimal concentration of GDC-0449 for teratogenic effects was 150 mg•kg^−^^1^ b.w. Exposure between E9.5 and 10.5 induced frontal bone dysplasia, micrognathia and limb defects. Due to the high rate of maternal death and embryonic lethality in the GD9.5 group, we chose the GD10.5 group as our model for subsequent research.

### Morphogenesis of the frontal bones in GD10.5 embryos

**Fig. 3:**
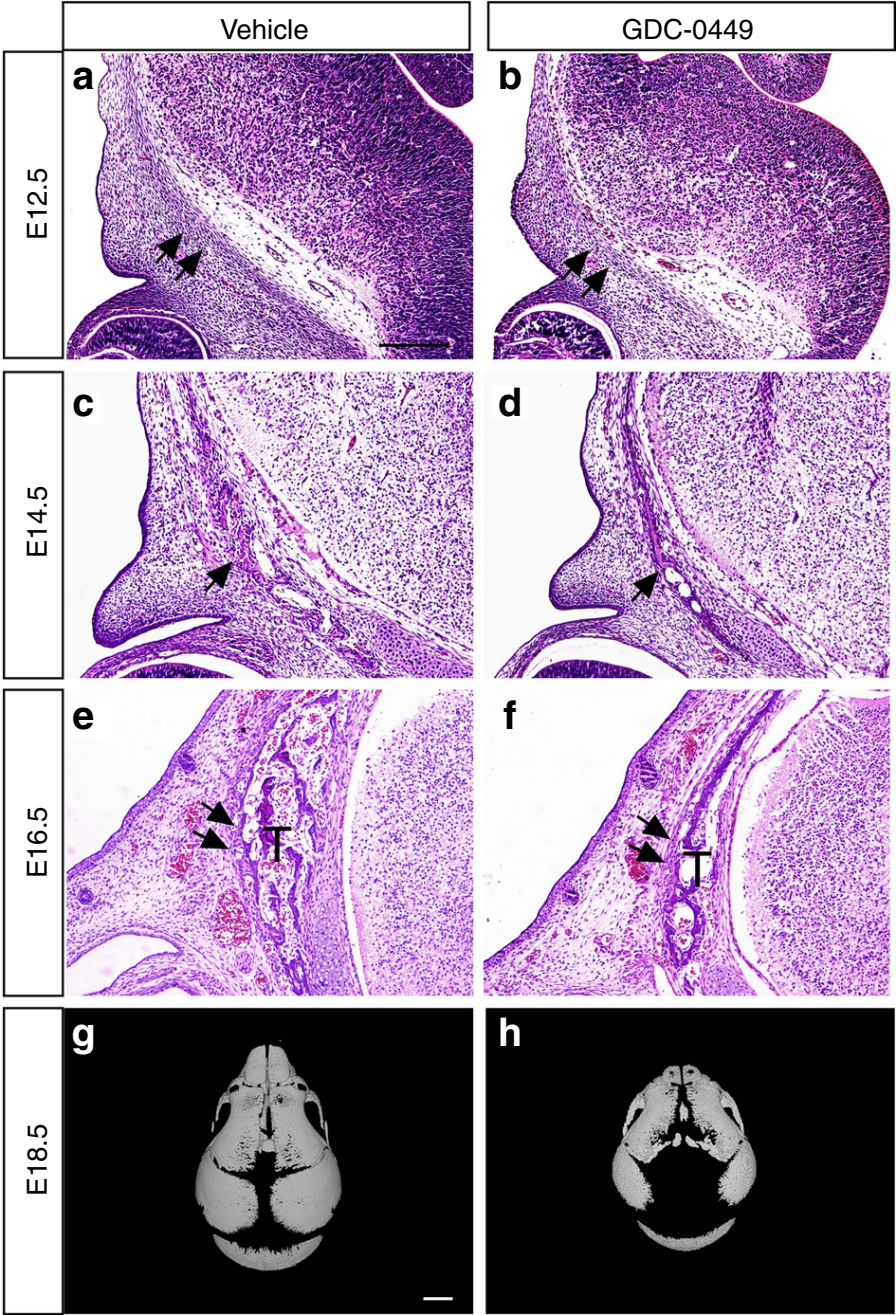
Frontal bone development in GD10.5 embryos. Histology of the frontal primordium from E12.5 (**a**, **b**), E14.5 (**c**, **d**), and E16.5 (**e**, **f**) embryos. **a**, **b** In GD10.5 embryos, a reduced cell density (double arrow) was observed in the frontal primordium at E12.5. **c**, **d** At E14.5, mineralized bone was formed within the frontal bone primordium, but there was less bone matrix (arrow) in GD10.5 embryos. **e**, **f** At E16.5, frontal bone formation was visible in the vehicle-exposed and GD10.5 samples (double arrow). In GD10.5 embryos, the frontal bones had a reduced trabecular structure (T) and reduced thickness. **g**, **h** 3D images generated from micro-CT scans of E18.5 calvaria. Relative to vehicle-exposed embryos, the frontal bones and parietal bones were smaller with a concomitant increase in the width of the metopic and sagittal sutures in GD10.5 mice. Scale bars = 200 μm in (**a–f**); 1 mm in (**g**, **h**)

**Fig. 4:**
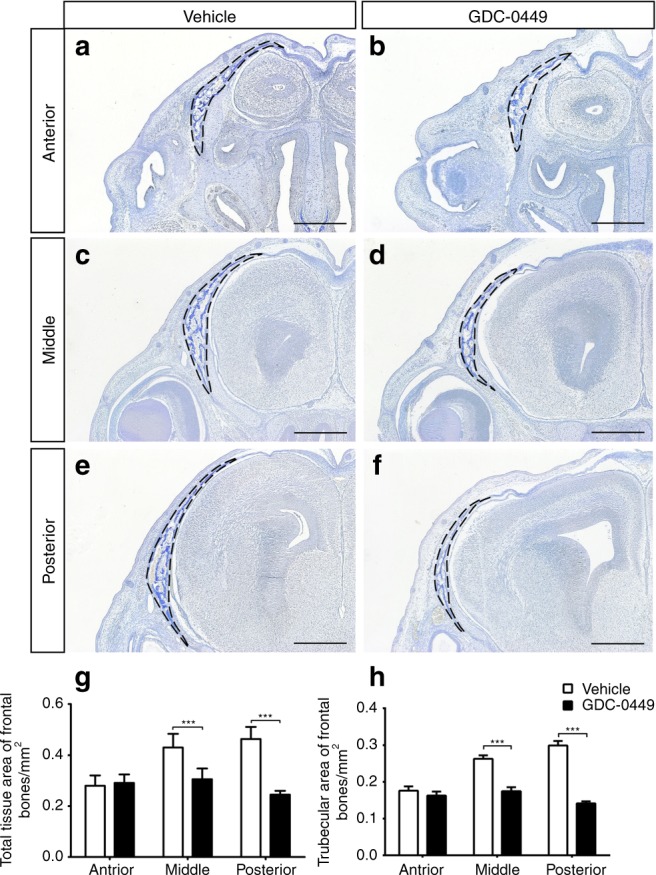
Compromised frontal bone formation in GD10.5 embryos at E16.5. Sections were cut through the anterior (**a**, **b**), middle (**c**, **d**), and posterior (**e**, **f**) portions of the eyes. In these Aniline blue-stained sections, the osteoid appears dark blue (dotted black lines). **g** Total tissue area of frontal bones. **h** Trabecular area of frontal bones. There was no obvious difference in the areas of the frontal bones or trabecular structure in the anterior portions of the frontal bones between vehicle-exposed and GD10.5 embryos. However, in GD10.5 embryos, the frontal bones were smaller and the trabecular structure considerably looser in the middle and posterior portions of the frontal bones. *n* = 6 embryos per group. Values are the mean ± SD. Data were analysed using unpaired Student’s *t* tests. ***P< 0.001. Scale bars = 500 μm

To the best of our knowledge, current research to uncover the aetiology of frontal bone dysplasia has largely utilized genetic abrogation. It is difficult to identify the function of Hh signalling in spatiotemporally dependent aspects of frontal bone development using such an approach. Thus, the aim of this study was to gain a more thorough developmental understanding of Hh signalling-dependent frontal bone development. To observe the development of the frontal bone primordium, we conducted a histological examination of GD10.5 and vehicle-exposed embryos from E12.5 to E16.5. At E12.5, the frontal bone primordium was initiated supraorbitally where the mesenchyme forms a condensation in vehicle-exposed mice (Fig. 3a). In GD10.5 embryos, we found reduced condensation of mesenchymal cells (Fig. 3b). At E14.5, the amount of bone matrix present in the frontal bone primordium was reduced in GD10.5 embryos compared with vehicle-exposed embryos (Fig. 3c, d). At E16.5, frontal bone formation was visible in both the vehicle-exposed and GD10.5 embryos. However, in GD10.5 embryos, the frontal bones were reduced in trabecular structure and reduced in thickness (Fig. 3e, f). To further investigate the differences in osteogenesis between vehicle-exposed and GD10.5 embryos, we cut serial coronal sections of the frontal bones and stained them with aniline blue (Fig. 4a–f). The frontal bones of vehicle-exposed mice extended towards the top of the calvaria where the ossification fronts were juxtaposed, separated by the metopic suture. There were no obvious differences in the area of the frontal bones or in the trabecular structure of the anterior portions of the frontal bones between vehicle-exposed and GD10.5 embryos (Fig. 4a, b, g, h). However, we detected significantly reduced formation of the frontal bones with a looser trabecular structure in the middle and posterior portions of the frontal bones of GD10.5 embryos (Fig. 4c–f, g, h). We generated 3D images of the calvaria based on micro-CT of E18.5 embryos and found that the frontal bones and parietal bones were smaller in GD10.5 mice, with a concomitant increase in the width of the metopic and sagittal sutures compared with those of vehicle-exposed mice (Fig. 3g, h). Taken together, our findings indicate that administration of Hh antagonist at E10.5 induced delayed formation and defective ossification of the frontal bone primordium.

### Downregulation of Hh signalling in GD10.5 embryos

**Fig. 5:**
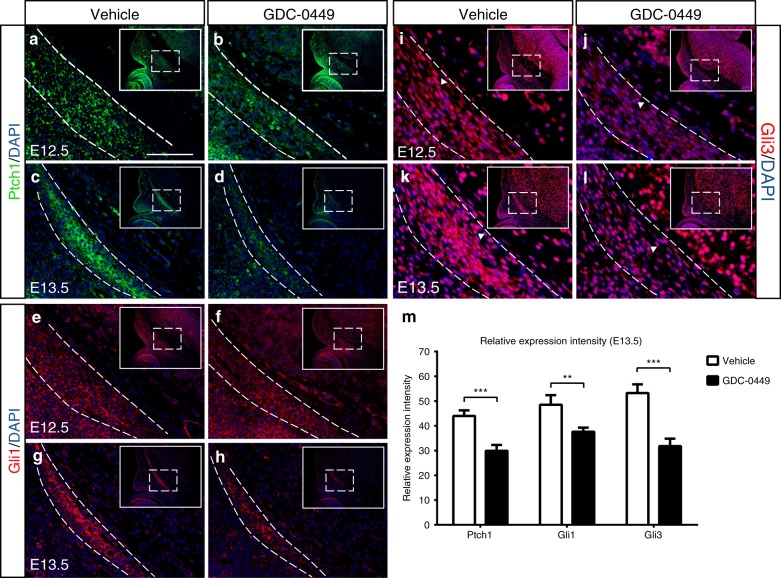
Expression of Hh signalling pathway effectors in embryos. At E12.5, compared to vehicle-exposed embryos, immunofluorescent staining for Ptch1 (**a**, **b**) and Gli1 (**e**, **f**) in coronal sections of GD10.5 embryos showed a slight reduction in the frontal bone primordium (dotted white lines), and an even greater decrease was observed in Gli3 expression (**i**, **j**) in the frontal bone primordium. At E13.5, Ptch1 (**c**, **d**), Gli1 (**g**, **h**) and Gli3 (**k**, **l**) expression levels were markedly diminished in the frontal bone primordium of GD10.5 embryos compared with vehicle-exposed embryos. Consistent with this finding, the sections in the white boxed areas show lower magnification views of the frontal bone primordium. Arrowheads point to Gli3-negative cells. **m** There was a significant reduction in p = Ptch1, Gli1 and Gli3 intensity in the frontal bone primordium in GD10.5 embryos in comparison to vehicle-exposed embryos (*n* = 6 embryos per group from three litters). The values are the mean ± SD. Data were analysed using unpaired Student’s *t* tests. **P< 0.01; ***P< 0.001. Scale bars = 50 μm

To assess whether Hh signalling was altered in the frontal bone primordium of GD10.5 embryos, immunofluorescence experiments were performed at E12.5. The results showed a slight reduction of Patched1 (Ptch1) and Gli1 expression levels in GD10.5 embryos compared with vehicle-exposed embryos (Fig. 5a, b, e, f), and Gli3 levels were further reduced in the frontal bone primordium (Fig. 5i, j). At E13.5, the expression of Ptch1 was significantly reduced in GD10.5 embryos. Gli1 had a similar expression pattern to that of Ptch1, and Gli1 protein expression was decreased in GD10.5 embryos (Fig. 5c, d, j, h). The expression level of Gli3 was also further reduced in GD10.5 embryos compared with vehicle-exposed embryos (Fig. 5k–l). These findings revealed a general downregulation of Hh signalling in GD10.5 embryos. Quantitation of the Ptch1, Gli1 and Gli3 intensity in serial sections through the supraorbital ridge confirmed this decrease (Fig. 5m).

### Inhibition of Hh signalling affects the migration of CNCCs into the frontal primordium

**Fig. 6:**
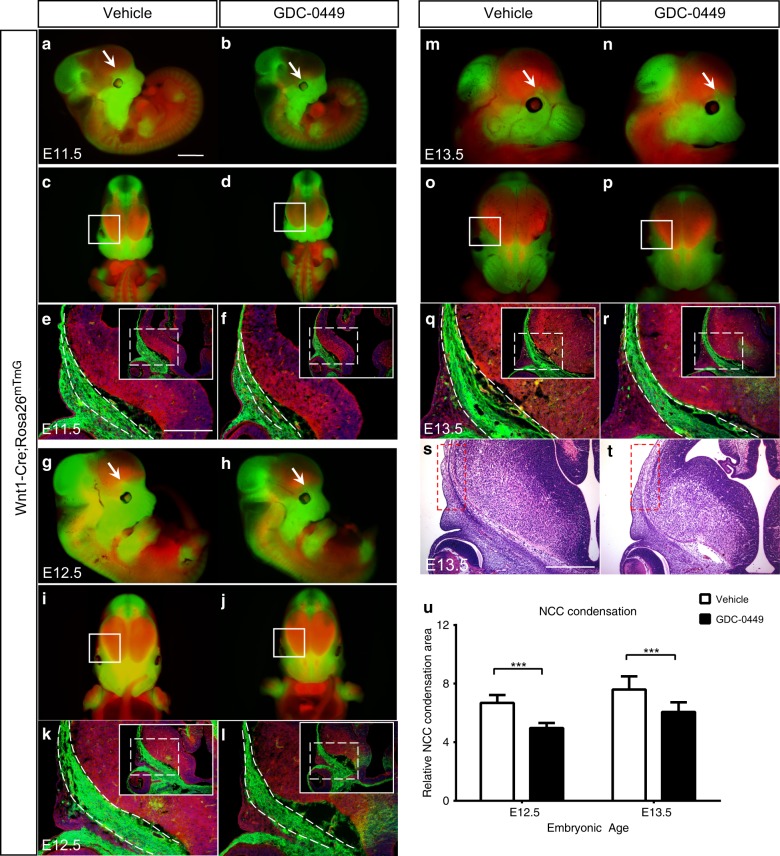
Migration of CNCCs in vehicle-exposed and GD10.5 embryos. Migration of transgene-expressing CNCCs in *Wnt1Cre; Rosa26*^*mTmG*^ embryos, as evidenced by Gfp expression, to their target destinations (arrow and pane) at E11.5 **a**–**f** E12.5 (**g**–**l**), and E13.5 (**m**–**r**). At E11.5 and E12.5, frontal (**a**, **b**, **g**, **h**) and lateral (**c**, **d**, **i**, **j**) views showed that the CNC-derived frontal bone condensation was smaller in GD10.5 embryos than in vehicle-exposed embryos, and it displayed a lack of Gfp expression. **m**–**p** At E13.5, the frontonasal region was shorter in GD10.5 embryos, resulting in less CNC-derived frontal primordium in the caudal-to-rostral direction. Cross-sections of E11.5 (**e**, **f**), E12.5 (**k**, **l**) and E13.5 (**q**, **r**) heads from GD10.5 and vehicle-exposed mice showed a reduction in Gfp expression in the GD10.5 frontal primordium compared with vehicle-exposed embryos. The insets show lower magnification views of the frontal bone primordium. **s**, **t** Histological analyses of vehicle-exposed and GD10.5 embryos at E13.5 showed a reduced extent of apical migration in GD10.5 embryos. The dotted box indicates the apical expansion of CNCCs. **u** A statistically significant decrease in the relative NCC condensation area at E12.3 and E13.5 was noted in the GD10.5 frontal bone primordium. Data were obtained from 12 sections of three pairs of control and mutant embryos. Values are the mean ± SD. Data were analysed using unpaired Student’s *t* tests. **P< 0.01; ***P< 0.001. Scale bars = 1 mm, except 500 μm in (**e**, **f**, **k**, **l**, **q–t**)

The frontal bones develop from CNCCs that initially condense in the presumptive frontal primordium prior to formation of the frontal bone. This is a vital process for normal frontal bone development.^4,17^ Thus, we conducted a lineage tracing experiment using *Wnt1-Cre*, a CNC-specific Cre transgene, and the double-fluorescent Cre reporter *Rosa26*^*mTmG*^ to label CNCCs. *Wnt1-Cre; Rosa26*^*mTmG*^ mice express tdTomato in non-recombined cells and green fluorescent protein (Gfp) in recombined cells.^18–20^ We observed Gfp expression in whole embryos of vehicle-exposed and GD10.5 samples at E11.5. Gfp-expressing CNCCs were primarily concentrated in the supraorbital ridge where the frontal primordium was developing in the vehicle-exposed embryos (Fig. 6a, c). There were subtle changes to the Gfp-expressing CNCCs in the presumptive frontal primordium in GD10.5 embryos (Fig. 6d, e). In the vehicle-exposed embryos, Gfp expression was detected in the caudal-to-rostral direction in the developing frontal bone region at E12.5 (Fig. 6g, i). There was a decrease in Gfp-expressing CNCCs in the frontal bone condensation in GD10.5 embryos at this stage (Fig. 6h, j). By E13.5, CNC-derived cells had migrated apically towards the top of the calvaria. A reduced extent of apical migration and a shorter frontonasal region were observed in GD10.5 embryos, resulting in less Gfp-expressing CNC-derived cells in the frontal primordium region than in the vehicle-exposed embryos (Fig. 6m–p). Furthermore, examination of tissue sections at the corresponding time points revealed a significant reduction in Gfp-expressing CNCCs in GD10.5 frontal primordium compared to the vehicle-exposed embryos (Fig. 6c, d, k, l, q, r). Similarly, by E13.5, histological analyses of vehicle-exposed and GD10.5 embryos showed a reduced extent of apical migration in GD10.5 embryos (Fig. 6s, t). Quantitation of the NCC condensation by counting the mesenchymal cell condensation area revealed significant reductions in the supraorbital ridge and top of the calvaria of GD10.5 embryos both at E12.5 and E13.5 (Fig. 6u). This result suggests that time-specific inhibition of Hh signalling in mice appears to affect the migration of CNCCs after E10.5, resulting in frontal bone hypoplasia.

### Cell proliferation diminishes and cell death increases in frontal bone condensation in GD10.5 embryos

**Fig. 7:**
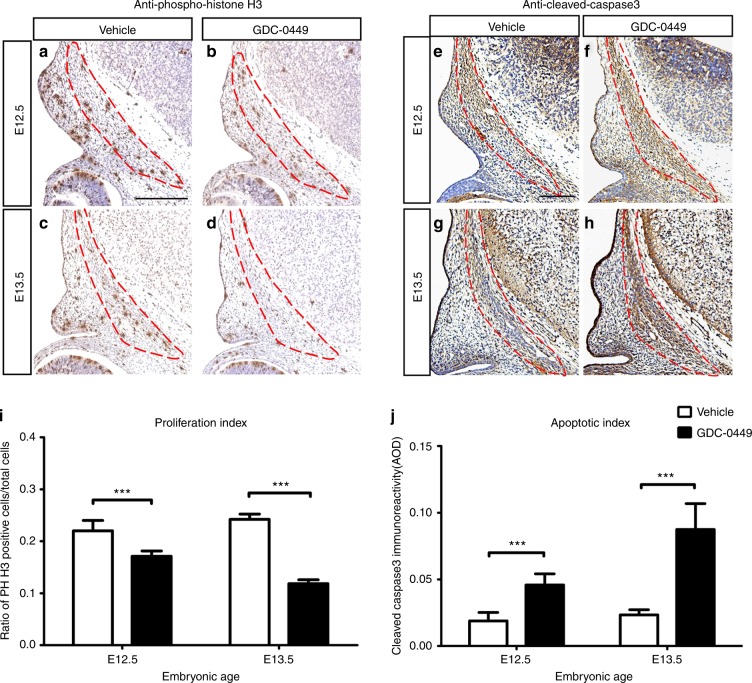
Reduced cell proliferation and increased cell death within the frontal primordium in GD10.5 embryos. **a–h** Red outlines indicate the frontal bone primordium. **a–d** Immunostaining with anti-phosphorylated histone H3 showed a significant decrease in cell proliferation in GD10.5 embryos. **e–h** Cell death was detected by antibody detecting cleaved caspase3. GD10.5 embryos showed increased cell death. **i** Statistical analysis of cell proliferation in the frontal bone primordium at E12.5 and E13.5. A significantly lower ratio of cells was observed in GD10.5 at both age stages. **j** A statistically significant increase in the apoptotic index at E12.5 and E13.5 was noted in the GD10.5 frontal bone primordium. *n* = 12 for each group at each age. The values are the mean ± SD. Data were analysed using unpaired Student’s *t* tests. ***P< 0.001. Scale bars = 200 μm

Preosteoblast differentiation and proliferation have been shown to begin at around E12 at the initial condensation of the frontal bones. While the differentiation proceeds apically at E12.5, the bone anlage continues its growth through the proliferation of preosteoblasts. The proliferation of these cells in the frontal bone rudiment also accounts for frontal bone growth.^21,22^ *Foxc1*^*ch/ch*^, a mutant mouse with a spontaneous loss of *Foxc1* function, displays decreased osteogenic cell proliferation in the E13.5 frontal bone primordium, which has been postulated to cause defects in the apical part of the skull vault.^23^ Analysis of cell proliferation and apoptosis in *Tgfb2* mutant mice showed that conditional inactivation of *Tgfbr2* in the neural crest lineage perturbs the proliferation, but not the survival, of cells in the frontal bone primordium from E12.5 to E14.5 during frontal bone development.^24^ As mitogens, hedgehog ligands regulate cell cycle genes across numerous cell types.^25^ Thus, we also considered the possibility that the smaller frontal bones in mice exposed to GDC-0449 were secondary to preosteoblast proliferation defects. We carried out immunochemical staining for phospho-histone H3 (PHH3) to detect the level of cell proliferation in the frontal bone primordium. At E12.5 and E13.5, we observed a significant reduction of cell proliferation activity in GD10.5 embryos compared with vehicle-exposed embryos (Fig. 7a–d, i). We also examined cell death by labelling with a cleaved caspase3 antibody, which revealed an increase in apoptotic activity in GD10.5 embryos compared with vehicle-exposed embryos (Fig. 7e–h, j). Taken together, our data suggest that the frontal bone defects observed in GD10.5 mice can be attributed to reduced cell proliferation and increased apoptosis in the frontal bone primordium.

### Osteogenic progenitor cell differentiation in the frontal bone primordium

**Fig. 8:**
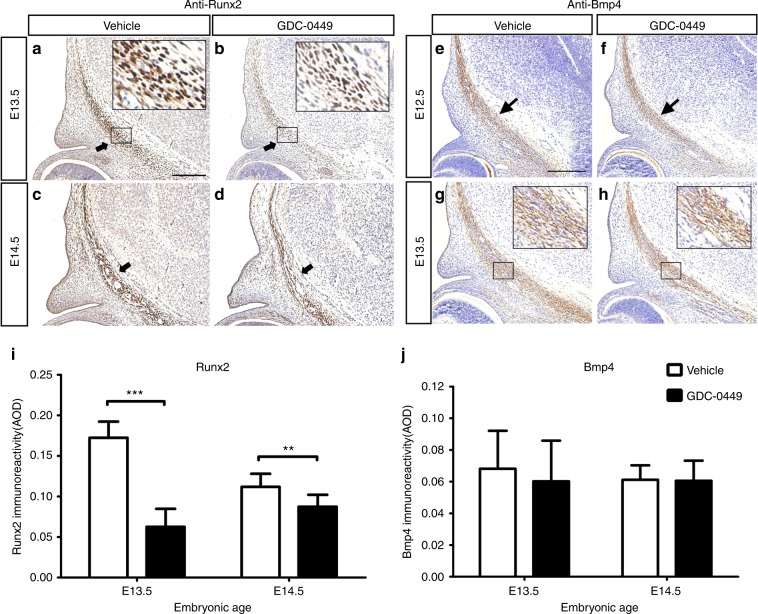
Osteogenic progenitor cell differentiation in the frontal bone primordium. Immunohistochemistry of Runx2 at E13.5 (**a**, **b**) and E14.5 (**c**, **d**) and Bmp4 at E12.5 (**e**, **f**) and E13.5 (**g**, **h**) in vehicle-exposed and GD10.5 mice. The insets show higher magnification images of the boxed areas. **a**–**d** At E13.5 and E14.5, expression levels of Runx2 in frontal bone primordium (black arrow) were lower in GD10.5 mice. **e**–**h** There was no obvious difference in the expression of Bmp4 between vehicle-exposed and GD10.5 mice. **i**, **j** Statistical analysis of relative expression levels of Runx2 and Bmp4 (*n* = 12 for each group at each age). Values are the mean ± SD. Data were analysed using unpaired Student’s *t* tests. **P< 0.01; ***P< 0.001. Scale bars = 200 μm

In mice, neural crest-derived osteogenic precursor populations combine into the rudiments of the calvarial bones, where their expression of early osteogenic markers such as alkaline phosphatase (ALP) and Runx2 can be detected between E12.5 and E13.5.^4,26,27^ After E13.5, migration and differentiation proceed in waves apically.^28^ It has been shown that disruption of Runx2 expression after E13.5 is responsible for the defects in the apical part of the frontal bone seen in *Foxc1*^*ch/ch*^ mice.^23^ Cyclopamine, a GDC-0449 analogue, has been shown to inhibit osteogenesis and Hh pathway activity in calvarial-derived cells in vitro.^29^ In a previous study, suture mesenchymal cells were collected and treated with Ihh or GDC-0449. Significantly upregulated Gli1 activity and increased osteogenic activity were found in the Ihh-treated cells, whereas significantly downregulated Gli1 activity and decreased osteogenic activity were found in the GDC-0449-treated cells.^30^ Our previous work has shown that disruption of Hh signalling by GDC-0449 delays ossification in the palatine bone region with downregulation of Ihh.^13^ In this study, GD10.5 embryos exhibited reduced Ptch1, Gli1 and Gli3 expression levels in the frontal primordium at E13.5 compared to vehicle-exposed embryos. To investigate whether a defect in osteoprogenitor differentiation during apical migration might have contributed to the frontal bone hypoplasia associated with wider metopic sutures in GD10.5 embryos, we examined the expression of Runx2 at E13.5 and E14.5. We found that the staining intensity and distribution of Runx2 were prominently reduced in GD10.5 samples at E13.5 and E14.5 (Fig. 8a–d, i).

Multiple studies have proposed that the Bmp signalling pathway plays a vital role in the formation of neural crest-derived calvarial bone.^6,31^ Bmp2 and Bmp4 are both downstream targets of Ihh in the developing calvaria,^29^ and murine in situ hybridization studies have shown that both Bmp2 and Bmp4 are expressed in the frontal primordia at E12.5 and E13.5.^32,33^ To assess whether Bmp4 signalling is responsible for Hh signalling in GD10.5 embryos, we carried out immunochemical staining to examine the expression of Bmp4, which showed no obvious difference in the frontal primordium between GD10.5 and vehicle-exposed samples (Fig. 8e–h, j).

## Discussion

Disruption of the Hh signalling pathway results in variety of craniofacial abnormalities in transgenic mouse models and clinical cases.^7,15,34^ However, genetic approaches using mutant mice are not able to pinpoint the critical periods of Hh signalling pathway involvement in skull development. Recognizing the critical periods of embryonic susceptibility to Hh pathway antagonists in calvarial development, as a complementary strategy to genetic approaches, is useful for understanding the complex developmental toxicity and aetiology, and provides a flexible way to investigate the mechanisms through which dysmorphogenesis can arise from chemicals. GDC-0449 is a low-molecular-weight inhibitor of the Hh signalling pathway that blocks the function of Smoothened, causing inactivation the Gli family of transcription factors.^10^ Here, we detected patterns of dysmorphology attributed to GDC-0449 exposure, including frontal bone dysplasia, micrognathia, cleft palate (data not shown) and limb defects, similar to those seen in mutant mouse models.^29,35,36^ Expression of Shh is essential for mandibular development, and loss of Shh in these domains leads to micrognathia resulting from increased mesenchymal cell death in pharyngeal arch 1 (PA1) after CNCC migration.^37^ Shh signalling is required for the outgrowth of the limb and for the development of structures distal to the elbow/knee in the mouse through specifying antero-posterior positional values during normal development in a concentration-dependent and time-dependent fashion.^38^ In this study, we used GDC-0449 to block the function of Hh. Exposed embryos exhibited micrognathia and limbs that tapered towards the tip with only one to three digit-like structures, similar to the phenotypes observed in mouse embryos lacking Shh function. In addition, our previous study has shown that disruption of Hh signalling by GDC-0449 leads to cleft palate and delayed osteogenesis in the mouse.^13^ However, in this study, we focused on the mechanisms by which Hh function is disrupted by GDC-0449 during the migration, proliferation and differentiation of CNCCs in the frontal primordium. We demonstrated that the critical period for exposure leading to these phenotypes was between E9.5 and E10.5, and the optimal teratogenic concentration delivered orally to the pregnant dam was 150 mg•kg^−^^1^ b.w. Mice administered GDC-0449 at embryonic days 9.5 or 10.5 (GD9.5 and 10.5 groups, respectively) had significantly smaller frontal bones associated with wider metopic sutures compared with controls (Figs. 1 and 2). Moreover, GD10.5 mice exhibited smaller frontal and parietal bones with a concomitant increase in the width of the metopic and sagittal sutures (Fig. 2). It would therefore be interesting to study the differences between the frontal bones and parietal bones, which are derived from different cell populations.^4,28,39^

Hh signalling plays a vital role in the development of craniofacial width.^40,41^ Following Hh ligand binding to Ptch1 to release Smoothened (Smo), Smo uses intraflagellar transport proteins (IFT) to translocate to the cilium and promote Gli transcription factor activation and then to further promote Hh target genes.^42^ Knocking out Hh-responsiveness specifically in CNCCs results in a severe defect in frontal bone development. *Ihh*-null allele mice show a reduction in cranial bone size and in all markers of osteodifferentiation.^9,29^ A newly isolated and characterized, N-ethyl-N-nitrosourea (ENU)-induced recessive mouse model (*Ptch1*^*DL*^) exhibits a widened interfrontal suture and heterotopic ossification, resembling the phenotype of the Gli3 loss-of-function mouse model (*Gli3* ^*Xt-*^^*J/Xt-J*^).^36,43^ In our model, levels of the Hh receptor Ptch1 as well as the transcription factors Gli1 and 3 were reduced (Fig. 5). Here, we showed that the Hh pathway antagonist GDC-0449 acts directly on Smo to reduce the occupancy of Ptch1 and thus restrict Gli1 and Gli3 expression to the frontal primordium fated to become the developing frontal bone. We propose that within the frontal primordium, Hh functions through Ptch1 to establish a threshold level of Gli transcription factors, thus regulating the proliferation and differentiation of osteogenic progenitor cells and the formation of calvarial bones. Our data demonstrate that the frontal bone defect observed in these mice results from the perturbation of Hh signalling.

The frontal bones develop exclusively from CNCCs, which migrate into the presumptive frontal primordium to form the skeletogenic mesenchyme condensations.^26,39^ Increased Hh activity due to truncation of the primary cilia on CNCCs leads directly to increased proliferation of CNCCs, which contributes to hypertelorism and frontonasal dysplasia.^40^ Through exogenous interference with the Hh pathway, we observed a decreased condensation of mesenchymal cells in the frontal bone primordium (Fig. 3a, b). Although Hh signalling has a significant role in craniofacial development as well as during endochondral ossification, Ihh expression was not detected in the heads of the studied mouse embryos by in situ hybridization until E12, which demonstrated that Shh was exclusively responsible for Hh signalling during the development of the head prior to E12.^44,45^ Shh plays an important role in the development of postmigratory CNCCs, and loss of Shh function in CNCCs results in defects in the frontal bones.^44^ *K14-Shh*, a transgenic mouse model that expresses Shh in basal epithelium under a Keratin-14 promoter, displays an absence of flat bones within the skull vault and truncated frontal bones, resulting from the sensitivity of CNCCs to abnormal Hh signalling levels.^46^ Ptch1 is expressed in the dense population of CNCCs present from E9.5 to E12.5, suggesting that direct Hh signalling may have functional importance for this condensation of cells, similar to the Ptch1 expression observed in the frontal primordia of our embryos.^44^ Therefore, we conducted a detailed analysis of the migration of CNCCs in *Wnt1-Cre; Rosa26*^*mTmG*^ mice, which showed reduced CNCC induction or migration in GD10.5 mice (Fig. 6). We propose, consistent with multiple studies,^40,44^ that Hh signalling is an essential regulator of CNCC induction, migration and population size during embryonic development. Frontal bone precursors, which are derived from CNCCs, first move in a caudal-to-rostral direction at E11.5 – E12.5, followed by apical migration at E13.5.^4^ At E12.5 and E13.5, GD10.5 embryos exhibited reduced Ptch1, Gli1 and Gli3 expression levels in the frontal primordium compared with vehicle-exposed embryos. We also suggest that the compromised condensation of the CNC-derived mesenchyme in the frontal bone primordium due to disruption of the Hh pathway at E10.5 is a trigger for the defect observed in early frontal bone development.

Increased proliferation of CNCCs can be directly caused by increased Hh activity.^25,40^ Loss of Ihh has been shown to reduce preosteoblast proliferation and delay calvarial osteogenesis, leading to smaller bones.^47,48^ Furthermore, ectopic expression of Shh in the frontonasal prominence (FNP) between stages 20 and 21 in the chick (equivalent to E10.5 and E11.5 of the mouse) leads to a mediolateral expansion of the FNP, which results from increased cell proliferation after addition of the protein.^49^ In another study, Hh signalling was removed in CNCCs and resulted in increased apoptosis and decreased cell proliferation in the branchial arches.^44^ In addition, our previous study has shown that during treatment with GDC-0449, the cell proliferation rate of CNC-derived palatal shelves is decreased, which likely contributes to the development of complete cleft palate.^13^ Thus, we also propose the possibility that the smaller frontal bones in mice treated with GDC-0449 are secondary to preosteoblast proliferation defects (Fig. 3e, f). Preosteoblast differentiation and proliferation begin around E12 at the initial condensation of the frontal bones.^21^ Our model exhibited reduced proliferation and increased apoptosis within the frontal primordium. This major mechanistic defect may be responsible for the frontal bone deficiency observed in GDC-0449-exposed embryos (Fig. 7). However, Ptch1 and Gli transcription factors were downregulated in migrating CNCCs, and no Hh ligands or their downstream targets were detected, which suggested that the observed reduction in proliferation and increase in apoptosis within the frontal primordium might not in fact be directly responsive to Hh signalling.

Ihh has been shown to positively regulate calvarial ossification and modulate BMP signalling during calvarial development.^29^ Cytology experiments have shown that GDC-0449-treated cells exhibit significantly downregulated Gli1 activity. This downregulation of Hh activity is followed by a decrease in osteogenic activity.^30^ Our previous work has indicated that disruption of Hh signalling by GDC-0449 delays ossification in the palatine bone region along with downregulation of Ihh protein.^13^ Our present findings suggested that frontal bone hypoplasia was induced mainly by alterations in CNCC migration and survival/proliferation in the frontal primordium. It was not clear whether inhibition of Hh function during the migration of CNCCs into the frontal primordium would also impact osteoblast differentiation. We hypothesized that the deficiency of bone matrix and late bone formation observed in GD10.5 embryos might result from a defect in osteogenic differentiation induced by the disruption of Hh signalling (Fig. 3c–h). Our results indicated that the reduced expression of Runx2 in GD10.5 embryos was similar to that observed in *Ihh*- null allele mice.^9,48^ Ihh promotes osteoblast differentiation by initiating Runx2 expression.^50^ Supporting these results, our previous study demonstrated a decrease in the expression of Ihh and osteogenesis markers within the CNCC-derived palate in GDC-0449-treated embryos.^13^ Additionally, Ptch1 and Gli transcription factors were downregulated in the frontal primordium of GD10.5 embryos. We therefore propose that Hh signalling plays an important role in regulating the differentiation of post-migratory CNCCs into the osteogenic lineage. Smaller frontal bones with widened sutures may be the result of perturbations in CNCC migration and the survival/proliferation of migrating CNCCs combined with defects in osteoblast differentiation, which are regulated by Hh signalling. The detailed molecular mechanisms that lead to frontal bone defects in GDC-0449-exposed embryos still require further in vivo and in vitro investigation.

The BMP signalling pathway is essential for the osteogenic differentiation of the CNC-derived mesenchyme to form the calvarial bones.^31,51^ Conditional deletion of *Bmp2, Bmp4* or *Bmp7* in CNCCs leads to severe calvarial bone growth defects.^52^ However, because most Bmp knockout mice die prior to the development of detectable craniofacial deformities, it has been difficult to investigate the effects of Bmp signalling on osteoblast differentiation.^53,54^ Others have shown that Bmp2 and Bmp4 are both downstream targets of Ihh in the developing calvaria.^29^ Given that Hh and Bmp signalling are both involved in the earliest step of frontal bone development, the frontal bone defect observed in GD10.5 embryos suggests that Bmp signalling may be compromised during this stage. In the present study, we mainly focused on the critical period for Hh pathway antagonist-induced frontal bone hypoplasia and how the Hh signalling pathway regulates bone formation and CNCC migration. Further studies are acquired to reveal the crosstalk between Bmp and Hh signalling pathways in our mouse model.

In conclusion, we have identified the critical period of susceptibility for GDC-0449-mediated teratogenesis in the mouse and optimized the dosage for inducing craniofacial abnormalities without excessive maternal or embryonic lethality. Using this optimized model, which we call GD10.5 herein, we have found that the Hh signalling pathway regulates the migration, proliferation and differentiation of CNCCs into the frontal primordium. Moreover, this tractable mouse model also provides valuable opportunities for further investigating the cellular and molecular mechanisms of craniofacial malformations, and it will have a profound impact on our understanding of candidate human disease genes and culpable environmental factors.

## Materials and methods

### Timed mouse mating and breeding procedure

The experiments were approved by the Institutional Animal Care and Use Committee of Fujian Medical University. Institute　of　Cancer　Research (ICR) mice were purchased from the Shanghai Laboratory Animal Center, China (license no. SCXK2012-0002) and housed under specific pathogen-free conditions in disposable, ventilated cages. *Wnt1-Cre* and *Rosa26*^*mTmG*^ mice were kindly provided by Fujian Key Laboratory of Developmental and Neuro Biology, College of Life Science, Fujian Normal University. We generated heterozygous *Wnt1-Cre; Rosa26*^*mTmG*^ mice by crossing *Wnt1-Cre* mice with *Rosa26*^*mTmG*^ mice. Total DNA was extracted from a 5-mm piece of mouse tail. PCR was performed using the following primers: *Wnt1-Cre*: forward primer: 5’-TGCCAGGATCAGGGTTAAAG-3’ and reverse primer: 5’-GCTTGCATGATCTCCGGTAT-3’, which resulted in a PCR band of 400 bp; *Rosa26*^*mTmG*^: mTmG#1: 5’-CTCTGCTGCCTCCTGGCTTCT-3’; mTmG#2: 5’-CGAGGCGGATCACAAGCAATA-3’; mTmG#3: 5’-TCAATGGGCGGGGGT

GGTT-3’ (resulting in bands of 330 bp for wild-type and two bands of 250 bp and 330 bp for heterozygotes). All mice were maintained in an ICR background. The animal room was maintained at 22 °C ± 2 °C with a relative humidity of 50% ± 5%. The photoperiod was 12 h of artificial light and 12 h of darkness. Two or three nulligravid female mice were mated with a single male overnight, and embryonic day 0.5 (E0.5) was defined as detection of a vaginal plug on the following morning.

### Drug exposure

A suspension of 400 µL GDC-0449 (Selleckchem, Houston, TX) was prepared in 0.5% methyl cellulose (Aladdin, Shanghai) and 0.2% Tween (Shenggong, Shanghai). Single doses of GDC-0449 (100 mg•kg^−^^1^ b.w. or 150 mg•kg^−^^1^b.w.) were administered by intra-gastric intubation (gavage) to groups of pregnant mice at the indicated time points, including E8.5, 9.5, 10.5, 11.5 and 12.5. For clarity, we refer to these groups as GD8.5–12.5. Controls were gavaged with an equivalent volume of vehicle (0.5% methyl cellulose [Aladdin, Shanghai] with 0.2% Tween [Shenggong, Shanghai]) at the same time points. Embryos were collected at E16.5 or E18.5 for morphological assessment.

### Skeletal staining

After killing, heads of embryos aged E16.5 were skinned and fixed in 95% or 100% ethanol for at least 1 week and then placed overnight in Alcian blue staining solution (1 volume glacial acetic acid, 4 volumes 100% ethanol and 150 µg•mL^−^^1^ of Alcian blue 8GX [Sigma]). They were then washed twice in 100% ethanol for one hour and subsequently cleared in 2% KOH for 2–4 h. Tissues were transferred to Alizarin Red staining solution (1% KOH, 75 µg•mL^−1^ Alizarin Red-S [Sigma]) for 4–10 h to stain the bones. Samples were then rinsed in 2% potassium hydroxide. Finally, they were cleared in 1:3 glycerol in 2% potassium hydroxide for eight hours and stored in a 1:1 solution.

### Gross and histological morphometry

Osteogenesis was quantified using Image-Pro Plus 6.0 (Media Cybernetic, WA). Stained bone and cartilage of vehicle-exposed and GDC-0449-exposed mice (*n* = 2 from each of three different litters) were imaged at equivalent magnifications for gross morphometry (Fig. 2). The length of the anterior and posterior metopic suture, length and width of the head, area of the frontal bones and area of the patent suture were measured using Image-Pro Plus 6.0. Because heads of embryos vary in size, to control for the difference between individuals, we also measured the proportion of the frontal bone in the frontal region (grey area in Fig. 2m). All calculations were performed by a single-blinded investigator and confirmed by a second independent investigator.

### Micro-computed tomography analysis

Heads of embryos aged E18.5 that had been administered GDC-0449 (150 mg•kg^−^^1^ b.w.) or vehicle at E10.5 were fixed in 10% buffered formalin and then transferred to 70% ethanol. Scanning of the skull was performed on a micro-computed tomography (micro-CT) system (Research Center of Stomatology, Guangzhou Medical University, Guangzhou, China) at a thickness of 12 mm, energy of 55 kV, and intensity (lCT40) of 145 mA, and reconstructed to produce three-dimensional (3D) images.^55^

### Lineage tracing

CNC lineage tracing was performed with *Wnt1-Cre; Rosa26*^*mTmG*^ heterozygous mice. PCR genotyping of the mice was performed on tail biopsies. Samples were observed under a fluorescence microscope (Olympus-IX71), fixed in 4% paraformaldehyde overnight at 4 °C, and then dehydrated in 30% sucrose/PBS solution overnight at 4 °C. Samples were embedded in OCT compound (Tissue-Tek, Sakura), transferred onto dry ice to solidify, and cryosectioned at 8 µm thickness using a cryostat (Microm HM550). Images were captured using a fluorescence microscope (Olympus-IX71) with filter settings for DAPI/GFP/ tdTomato and merged with Adobe Photoshop CS6.

### Histology and immunohistochemistry

Embryos were fixed in 4% paraformaldehyde (PFA), followed by embedding in paraffin, Aniline blue staining and haematoxylin-eosin staining. For immunofluorescence, antibodies were obtained and diluted as follows: anti-Ptch1 (1:200, Abcam), anti-Gli1 (1:400, Abcam) and Gli3 (1:300, Abcam). Alexa Fluor® 488 Goat anti-rabbit and Alexa Fluor® 594 donkey anti-rabbit secondary antibodies were purchased from Life and used at a dilution of 1:200. For immunohistochemistry, antigen retrieval was performed, and sections were incubated with 3% H_2_O_2_ for 10 min to block nonspecific peroxidase activity. The primary antibodies used were as follows: anti-Runx2 (1:500, Abcam), anti-Bmp4 (1:300, Abcam), anti-cleaved caspase3 (1:1 000, Cell Signaling Technology) and anti-pH3 (1:1 000, Abcam). The secondary antibody was peroxidase-conjugated streptavidin goat anti-rabbit IgG (ready-to-use, ZSGB-Bio, Beijing). Staining was completed by incubation with diaminobenzidine, and counterstaining was performed with haematoxylin. To quantify Ptch1, Gli1 and Gli3 expression, six sections of three pairs of control and GD10.5 embryos were photographed and analysed using Image-Pro Plus 6.0 (Media Cybernetic, WA). Twelve sections from three pairs of control and GD10.5 embryos were stained with haematoxylin–eosin, and NCC condensation was measured by counting in the mesenchymal cell condensation area with Image-Pro Plus 6.0. The average optical densities (AODs) of Runx2 and Bmp4, cell proliferation and death were analysed using Image-Pro Plus 6.0 (Media Cybernetic, WA). Samples (*n* = 4) were obtained from each of three different litters treated with GDC-0449 at 150 mg•kg^−^^1^ b.w. or vehicle at E10.5.

### Statistical analysis

Statistical analysis was performed using SPSS version 19.0 for Windows. Data are expressed as the mean ± standard deviation (SD). Comparison of means between two groups was performed using the Student’s *t*-test. Differences between multiple groups were compared using one-way ANOVA. *P* < 0.05 was considered statistically significant.
